# Size distribution of extracellular vesicles in pretreatment ascites and plasma is correlated with primary treatment outcome in advanced high-grade serous carcinoma

**DOI:** 10.1038/s41598-025-88707-9

**Published:** 2025-02-06

**Authors:** Maruša Herzog, Ivan Verdenik, Borut Kobal, Katarina Černe

**Affiliations:** 1 Division of Gynecology and Obstetrics, University Medical Centre Ljubljana, 1000 Ljubljana, Slovenia; 2 Department of Gynecology and Obstetrics, Faculty of Medicine, University Ljubljana, 1000 Ljubljana, Slovenia; 3 Institute of Pharmacology and Experimental Toxicology, Faculty of Medicine, University of Ljubljana, 1000 Ljubljana, Slovenia

**Keywords:** High-grade serous carcinoma, Extracellular vesicles, Biomarkers, Nanoparticle tracking analysis, Resection success, Chemotherapy response, Treatment outcome, Cancer, Biomarkers

## Abstract

To improve the treatment outcome and survival of patients with advanced high-grade serous carcinoma (HGSC), prognostic biomarkers for assessing the feasibility of complete (R0) or optimal (R1) primary cytoreductive surgery are needed. Additionally, biomarkers for predicting the response to neoadjuvant chemotherapy (NACT) in patients with primary inoperable disease could help stratify patients for tailored therapy and improve personalised approach. Such promising biomarkers are extracellular vesicles (EVs), which are present in ascites and plasma and are available for minimally invasive liquid biopsy. EV concentration and EV molecular profile have been at the forefront of research in the field of biomarkers for many years now, but recent studies have highlighted the importance of EV size distribution. Our study aimed to evaluate the potential of the EV concentration and size distribution in pretreatment ascites and plasma samples from patients with advanced HGSC as prognostic biomarkers. In our prospective cohort study, nanoparticle tracking analysis (NTA) was used to determine EV characteristics in paired pretreatment ascites and plasma samples from 37 patients with advanced HGSC. Patients were treated with primary cytoreductive surgery followed by adjuvant chemotherapy (ACT) (N = 15) or NACT followed by interval debulking surgery (IDS) when optimal cytoreduction was not feasible (N = 22). The correlations of the EV concentration and size distribution in ascites and plasma with treatment outcome, progression-free survival (PFS) and overall survival (OS) were analysed. We found a significant correlation between the EV size distribution in ascites and residual disease after primary cytoreductive surgery. Larger EVs in ascites correlated with worse resection success after primary cytoreductive surgery. A significant correlation between the D10 value of EVs in plasma and the chemotherapy response score (CRS) after NACT was observed. A smaller D10 value of plasma EVs was correlated with a better chemotherapy response. Receiver operating characteristic (ROC) curve analysis revealed excellent performance for D10 value in ascites for the prediction of suboptimal (R2) resection at primary debulking surgery and excellent performance for D10 value in plasma for the prediction of complete or near-complete chemotherapy response score (CRS 3) at interval debulking surgery. There was a significant correlation between the mean diameter, D90 value and proportion of medium/large (> 200 nm) EVs in ascites and those in plasma. On the other hand, there was no correlation of the EV concentration or D10 and D50 values between the ascites fluid and plasma samples. Our results indicate that the EV size distribution in ascites has the potential to predict resection success after primary cytoreductive surgery and that the EV size distribution of the smallest EVs in plasma might help predict the chemotherapy response of patients treated with NACT. In the future, molecular analyses of size-dependent EV cargo could provide more insight into their biological functions and potential as predictive biomarkers.

## Introduction

High-grade serous carcinoma (HGSC) usually presents late with unspecific symptoms, and most cases are still diagnosed at an advanced stage, defined by the spread of the disease outside the pelvis [International Federation of Obstetrics and Gynaecology (FIGO) stage III and IV]^1–3^. The standard treatment for advanced HGSC is primary cytoreductive surgery followed by adjuvant chemotherapy (ACT) based on carboplatin and paclitaxel^1^. Most patients with HGSC initially respond to platinum-based chemotherapy but eventually develop chemoresistance, which makes recurrent disease incurable^1^. Thus, complete resection (R0) of all macroscopic disease thus still remains the single most important independent prognostic factor in advanced HGSC^1,4^. When complete or optimal (residual disease ≤ 1 cm, R1) cytoreduction with primary surgery is not expected, neoadjuvant chemotherapy (NACT) followed by interval debulking surgery (IDS) is proposed^1^. Currently, a laparoscopy-based Fagotti score is used for assessing the chance for optimal cytoreduction^5^. Despite advances, there remains a pressing need for better biomarkers to guide and stratify surgical care^6^. Prognostic biomarkers for tumour resectability in advanced HGSC that could help identify patients unsuitable for PDS without exposing them to the risk of diagnostic operation would enable earlier start of NACT and improve treatment outcome. While poly-ADP ribose polymerase (PARP) inhibitors have revolutionized treatment in patients with *BRCA*/homologous deficiency status, they complement rather than replace the need for optimal cytoreductive surgery^7^. HGSC containing a germline *BRCA* mutation often have better response to platinum-containing adjuvant chemotherapy compared to tumours with germline *BRCA* wild type. In contrast, recent study from Morgan *et.al.* reported that germline BRCA genotype was not associated with a superior histopathological response to NACT (CRS, chemotherapy response score). Thus, further research is needed to elucidate how the biology of HGSC influences CRS^8^. The development of composite biomarkers targeting platinum sensitivity could improve patient stratification and support more tailored, effective NACT.

Extracellular vesicles (EVs), present in all body fluids and accessible through liquid biopsy, represent a promising component of composite prognostic biomarkers for achieving more accurate primary treatment outcome^9^.Their release is increased in different pathological states, including cancer, and their cargo (proteins, lipids and nucleic acids) reflects the cell of origin^9,10^. In ovarian cancer, in vitro studies have shown that EVs play a role in tumour growth, metastasis and the development of chemoresistance^10–14^. There are, however, only a few clinical studies on the potential of EVs as biomarkers in ovarian cancer, most of which focus on diagnosis^15–17^.

Ascites is highly attractive as a source for biomarker discovery study. It is present in most (> 90%) patients with advanced HGSC and is available for minimally invasive liquid biopsy^18^, and studies have shown that EVs initially present in local fluid eventually become systemic and can be isolated from peripheral blood^19^. EV concentration and molecular profile have been at the forefront of research for many years now, but recent studies have highlighted the importance of EV size distribution^20–23^. Assessing whether changes of EV concentration and size distribution within the local environment can be detected through a blood test would be valuable.

Badovinac et al. performed a study on patients with pancreatic ductal carcinoma (PDAC) who underwent surgery with curative intent and reported higher EV concentration and smaller mean EV diameter in the plasma of patients who underwent resection than in those with primary inoperable disease. They concluded that preoperative plasma EV characteristics have potential as biomarkers for the resectability and overall survival (OS) of patients with PDAC ^23^. Our research group recently performed a study in which EV characteristics from a chemoresistant cell line were compared with those of two chemosensitive cell lines. We observed that the chemoresistant cell line released a more heterogeneous EV population and a higher proportion of medium/large sized (> 200 nm) EVs^24^.

Our study aimed to evaluate the potential of EV concentration and size distribution in pretreatment ascites and plasma samples from patients with advanced HGSC as prognostic biomarkers for primary treatment outcome. This is crucial, as it provides the best chance to eliminate cancer cells before they can adapt and develop resistance. Nanoparticle tracking analysis (NTA) was used to determine the EV concentration and size distribution in paired plasma and ascites samples. We found a significant correlation between the EV size distribution in ascites and residual disease after primary cytoreductive surgery. Additionally, a significant correlation between the D10 value of EVs in plasma and the chemotherapy response score (CRS) after NACT was observed. The EV concentration in plasma was significantly higher than that in ascites, and no correlation was found; however, there was a significant correlation between the mean D90 and the proportion of medium/large (> 200 nm)-sized EVs in ascites and those in plasma.

## Materials and methods

### Study design

Patients meeting the criteria of suspected or confirmed advanced HGSC were considered for participation in this prospective cohort study. The primary surgical procedures were carried out at the Gynaecological Department of University Medical Centre Ljubljana from October 2016 to August 2020. The baseline patient data included age, menopausal status, body mass index (BMI), American Society of Anaesthesiologists (ASA) score and preoperative CA125 tumour marker levels. Blood samples were taken on the day of the planned primary surgery, and ascites samples were taken at the beginning of the operation. In cases where optimal cytoreductive surgery was deemed feasible, primary debulking surgery (PDS) was conducted. The extent of resection was classified as complete (R0) with no macroscopic residual disease, optimal (R1) with less than 1 cm, and suboptimal (R2) with more than 1 cm of residual disease after cytoreductive surgery. If optimal cytoreduction was not anticipated, only diagnostic laparoscopy was performed. The pathology report included the residual disease status (R0, R1 or R2) and histological type of carcinoma. When the diagnosis of advanced-stage HGSC was not confirmed at primary surgery and/or with routine histopathological examination, patients were excluded from the study.

Patients who had PDS received adjuvant chemotherapy (ACT), while patients with primary inoperable disease were treated with neoadjuvant chemotherapy (NACT), which was succeeded by interval debulking surgery (IDS). When the patient’s general condition was too poor, only palliative chemotherapy was administered. The standard ACT/NACT regimen for HGSC is typically based on carboplatin in combination with paclitaxel; in cases where the patient’s general condition precluded this combination, carboplatin as monotherapy was administered. Bevacizumab, an antivascular endothelial growth factor (anti-VEGF) antibody, was added to the systemic therapy if the patient’s general condition and possible concomitant diseases allowed. Following the approval of the first PARP inhibitor, olaparib, as a first-line maintenance treatment on December 17, 2018, all patients with BRCA 1/2 mutation in our study were treated with olaparib in accordance with European Society for Medical Oncology (ESMO) guidelines applicable at the time. Homologous recombinant repair deficiency (HRD), acknowledged as a predictive biomarker for response to PARP inhibitors, was officially incorporated into the ESMO guidelines on December 1, 2020, after the conclusion of our study^25^. Consequently, not all patients who could benefit from PARP inhibitors were identified, likely impacting treatment outcome, particularly the duration of progression-free survival (PFS).

Patients who underwent IDS received a secondary pathology report that included the chemotherapy response score (CRS), which evaluates the response to chemotherapy (CRS 1 – minimal to no response; CRS 2 – partial response; and CRS 3 – complete or near-complete tumour response)^26^. CA125 values were monitored during and after chemotherapy for all patients, and the CA125 elimination rate constant K (KELIM score) was determined using the CA125 kinetics throughout the initial 100 days of chemotherapy. The patients’ vital status was recorded on August 1, 2023, with any missing data being explicitly denoted. Prior to study enrolment, all patients provided written informed consent, and the research adhered to the principles of the Declaration of Helsinki and was approved by the Republic of Slovenia National Medical Ethics Committee (KME 144/12/14). This study aligns with our previously published research where flow cytometry was utilized to assess total and EpCAM-positive EVs in ascites and plasma to evaluate their potential as biomarkers for advanced HGSC^27^.

### Sample collection

We collected samples from fasted individuals (for at least 12 h), which ensured that the blood was free of chylomicrons. Blood samples were drawn on the day of planned surgery with a 21-gauge needle. The first millilitres were used for routine clinical analyses. Next, 1.8 mL of blood for EV analysis was collected in BD vacutainer® Citrate blood collection tubes with 3.2% buffered sodium citrate solution. Ascites samples were taken at the beginning of the operation so that 50 ml was aspirated into a sterile syringe and then transferred into a sterile conical tube. The maximum permissible time between sample collection and preparation was 30 min.

### Sample preparation and storage

Blood and ascites samples were prepared following a protocol published by the International Society on Thrombosis and Haemostasis (ISTH), known informally as the “ISTH protocol”^28^. The ISTH protocol is the most widely used protocol to prepare plasma for EV research and was also endorsed by the American Heart Association^29^. All samples were processed by centrifugation in two stages at 2500×*g* for 15 min at room temperature to remove cells and debris. After each centrifugation, the supernatant was collected from at least 10 mm above the pellet. This process produces platelet-poor plasma (PPP) samples with less than 10^4^ platelets per μL. Since ascites may also contain platelets, this procedure was also applied to ascites samples. The depletion of platelets and the absence of haemolysis in the samples were verified using standard clinical laboratory tests ^29^. In EV research, complete cell removal from samples is essential to prevent cell-disruptive processes, such as freeze–thaw cycles, from generating cell fragments that could interfere with EV characterization. Additionally, the removal of all cells is important to avoid cell activation and postcollection EV release^30^. The plasma and ascites supernatants were then frozen in nitrogen vapour following a modified procedure for freezing sperm for artificial insemination^31^ and stored at − 80 °C until further purification for nanoparticle tracking analysis (NTA). The samples were allowed only one freeze–thaw cycle.

### Sample purification for nanoparticle tracking analysis in scatter mode (S-NTA)

Size exclusion chromatography (SEC; IZON qEV original/70 nm) was used to separate EVs from other particles. In addition to good recovery of EVs with almost complete removal of contaminants, this method is suitable for the purification of EVs from clinical samples because it is fast and simple, which is a basic requirement for clinical procedures^32–35^. Thawed samples (0.5 ml) were loaded and allowed to run into the column. Immediately after, Dulbecco’s phosphate-buffer saline (DPBS, without Ca^2+^ and Mg^2+^) was added. The first 3 ml of void volume was discarded, and then the next 1.5 ml, representing the zone where EVs are typically eluted, was collected. The elution of plasma and ascites protein is slower, eluting predominantly from 2.5 ml after the void volume. The optimal recovery size of EVs with this type of column is between 70 and 1000 nm, and with the exclusion of particles < 70 nm, the majority of contaminating lipoproteins are removed^36^. However, due to the possibility that some lipoprotein particles remained present (especially those larger than 70 nm), additional controls were used. We evaluated the influence of lipoproteins on our results by measuring the concentration of lipoproteins in our platelet-poor plasma (PPP) and ascites samples. Particle-enhanced immunonephelometry (Atellica® NEPH 630 System, Siemens Healthineers, Erlangen, Germany) was used to determine the concentrations of apolipoprotein A1 (ApoA1; HDL) and apolipoprotein B (ApoB; chylomicrons, VLDL, IDL, and LDL). No significant correlation between ApoA1 or ApoB levels in PPP or ascites samples before the procedures for lipoprotein exclusion and EV characteristics in plasma or ascites was found. After sample purification with SEC, the levels of ApoA1 and ApoB were below the detection limit in all the plasma and ascites samples. Given that blood contains high levels of free proteins and protein aggregates, we addressed the potential impact of these contaminating proteins on our results as well. The protein concentration was measured using the Bio-Rad protein assay following the Bradford method. After sample purification with SEC, the protein levels were below the detection threshold in all the samples. Since proteins are generally smaller than EVs,SEC allows for the effective separation of proteins from EVs. Additionally, the total protein content prior to purification was not correlated with EV characteristics in any sample (all *p* > 0.05).

### Quantification of EV size and concentration

EV size and concentration were determined by nanoparticle tracking analysis in scatter mode (S-NTA) using the NanoSight NS300 instrument (488 nm laser) connected to an automated sample assistant (both Malvern Panalytical, Worcestershire, UK). The samples were diluted to a concentration allowing accurate particle tracking (according to the manufacturer, app. 20–100 particles in the field of view). Each sample was measured four times for 80 s acquisitions at 25 °C and processed using NTA software (version 3.3). The number and duration of video captures were optimized to also detect larger EVs (> 200 nm) that were less abundant in our samples. A longer capture duration allows sufficient particles to be recorded, but excessive time should also be avoided, as it could lead to decreased concentration measurements due to particle adherence or sedimentation^37^. The particle size calculations and measurement temperature are linearly related, with the NTA software modal size analysis conducted between 20 °C and 27 °C aligning best with the expected size of 100 nm polystyrene nanospheres. Temperature control tends to be more challenging for ambient conditions compared to slightly elevated temperature. Therefore, the NanoSight NS300 default measurement temperature was set at 25 °C^38^. The camera level was adjusted according to the sample. The following settings were maintained for all the readings: detection threshold 5, water viscosity, automatic blur size and automatic (10.2–15.7 pix) maximum jump distance. The data obtained included the particle concentration (number of particles/ml of cell culture-conditioned media), mode (nm), mean (nm) and percentile values: D10, D50, and D90. The particle size values indicate that 10%, 50% and 90%, respectively, of the distribution was below this value. Reference nanospheres (100 nm polystyrene, Malvern Pananalytical, #LT3100A) were analysed on the same day to compensate for day-to-day variation in instrument performance. The raw data were analysed by NanoSight NTA 3.3 software (Malvern Panalytical, Worcestershire, UK). To calculate the concentration of particles for each sample, the dilution factor used during sample preparation (including a dilution factor of 3.4 when the sample passed through the qEV column) was considered. Data obtained were particle concentration (number of particles/mL of cell-cultured conditioned media), mode (nm), mean (nm) and size distribution defined as percentile values: D10, D50 and D90. Percentile values indicated that 10, 50 and 90%, respectively, of the particle distribution was below this value. The MISEV 2023 guidelines, prepared by ISEV, recommended for the first time that size distribution should also be reported, rather than just summary metrics such as mean, mode or median EV size, which can be skewed depending on the limit of detection and the asymmetry of the size distribution^30^.

To evaluate the suitability of our protocol for analysing EVs in clinical samples, our previous study involved analysing cell media derived from three different ovarian cancer cell lines^24^. In that study, NTA measurements were validated using transmission electron microscopy (TEM) and fluorescence-triggered flow cytometry (FT-FCM). Our findings indicated that, in addition to EpCAM-specific EVs, the distribution of EV sizes is a significant feature that might reflect the disease state^24^.

We have submitted all the relevant data from our experiments to the EV-TRACK knowledgebase (EV-TRACK ID: EV240145).

### Statistical analysis

All statistical analyses were conducted using IBM SPSS Statistics, version 29 (IBM Corporation, Armonk, NY, USA). Continuous variables were described using the mean, median, and interquartile range (25–75%). Categorical variables were described using frequencies. Pearson’s and Spearman’s Rho correlation coefficients were used to assess correlations between continuous variables. Receiver operating characteristic (ROC) curve analysis was performed, and the area under the curve (AUC) was calculated to evaluate the biomarker potential of different EV characteristics. The optimal cut-off values for specific EV characteristics in ascites and plasma were identified using the Youden index in ROC curves. Proportional Cox survival regression was used to assess progression-free survival (PFS) and overall survival (OS). PFS was defined as the time from primary surgery to disease recurrence, and OS was defined as the time from primary surgery to death from any cause. The Kaplan‒Meier test was used to compare PFS and OS between patients with PDS and patients with primary inoperable disease. All the statistical tests were two-sided, with the significance level set at 0.05.

## Results

### Patient demographic and treatment characteristics

The demographic and treatment characteristics of the 37 patients with advanced HGSC are presented in Table 1.

**Table 1:** Patient demographic and treatment characteristics.

Variables		Primary cytoreductive surgery	Primary inoperable	All patients
Number of cases		15	22	37
Age	Years, median (25–75%)	62 (57–76)	67 (60–73)	65 (59–74)
ASA score	1, *n* (%) 2, *n* (%) 3, *n* (%)	0 7 (46.7%) 8 (53.3%)	0 12 (54.4%) 10 (45.5%)	0 19 (51%) 18 (49%)
FIGO stage	IIIA, n (%) IIIB, n (%) IIIC, n (%) IVA, n (%) IVB, n (%)	1 (6.7%) 2 (13.3%) 11 (73.3%) 0 1 (6.7%)	0 0 19 (86.4%) 1 (4.5%) 2 (9.1%)	1 (2.7%) 2 (5.4%) 30 (81%) 1 (2.7%) 3 (8.1%)
Primary surgery	R0 R1 R2 inoperable	5 (33.3%) 7 (46.7%) 3 (20%) N/A	N/A N/A N/A 22 (100%)	5 (13.5%) 7 (18.9%) 3 (8.1%) 22 (59.4%)
Chemotherapy	Yes, ACT Yes, NACT + /- ACT Yes, paliative No	15 (100%) N/A N/A N/A	0 19 (86.4%) 2 (9.1%) 1 (4.5%)	15 (40.5%) 19 (51.4%) 2 (5.4%) 1 (2.7%)
Bevacizumab	Yes, n (%)	8 (53.3%)	8 (36.4%)	16 (43.2%)
PARPi	Yes, n (%) First-line, n (%) Second-line, n (%)	8 (53.3%) 4 (26.7%) 4 (26.7%)	0 0 0	8 (21.6%) 4 (10.8%) 4 (10.8%)
CA125 preoperatively	kU/l, median (25–75%)	658 (477–1047)	823 (447–2170)	758 (460–1825)
CA125 normalisation	Yes, n (%) Days, median (25–75%)	13 (86.7%) 64 (48–112)	14 (63.6%) 64 (40–103)	27 (73%) 71 (43–108)
KELIM score	< 1, n (%) ≥ 1, n (%)	12 (80%) 3 (20%)	17 (81%) 4 (19%) *	29 (80.6%) 7 (19.4%)
Disease progression/relaps	Early < 12 months Late ≥ 12 months or none	6 (40%) 9 (60%)	14 (63.6%) 8 (36.4%)	20 (54.1%) 17 (45.9%)
PFS	Months, median (25–75%)	13 (4–29)	9 (6–14)	10 (5–22)
OS	Months, median (25–75%)	25 (19–59)	32 (13–41)	29 (19–56)

*ASA* American Society of Anaesthesiologists, *FIGO* International Federation of Gynaecology and Obstetrics, *R0* complete resection (R0), *R1* optimal resection, *R2* suboptimal resection, *ACT* adjuvant chemotherapy, *NACT* neoadjuvant chemotherapy, *PARPi* poly-ADP ribose polymerase inhibitors, *KELIM* CA125 elimination rate constant K, *PFS* progression-free survival, *OS* overall survival.

^*^One missing case.

### Correlations of EV measurements between ascites and plasma

We used nanoparticle tracking analysis (NTA) to determine the concentration and size distribution of EVs in paired ascites and plasma samples. The concentration in ascites was 1.41 × 10^10^ (9.03 × 10^9^–2.49 × 10^10^) EVs/mL, and that in plasma was 2.64 × 10^10^ (1.92 × 10^10^–3.65 × 10^10^) EVs/mL. The concentration of EVs in plasma was significantly higher (p < 0.001) than that in ascites, but there was no significant correlation.

**Fig. 1:**
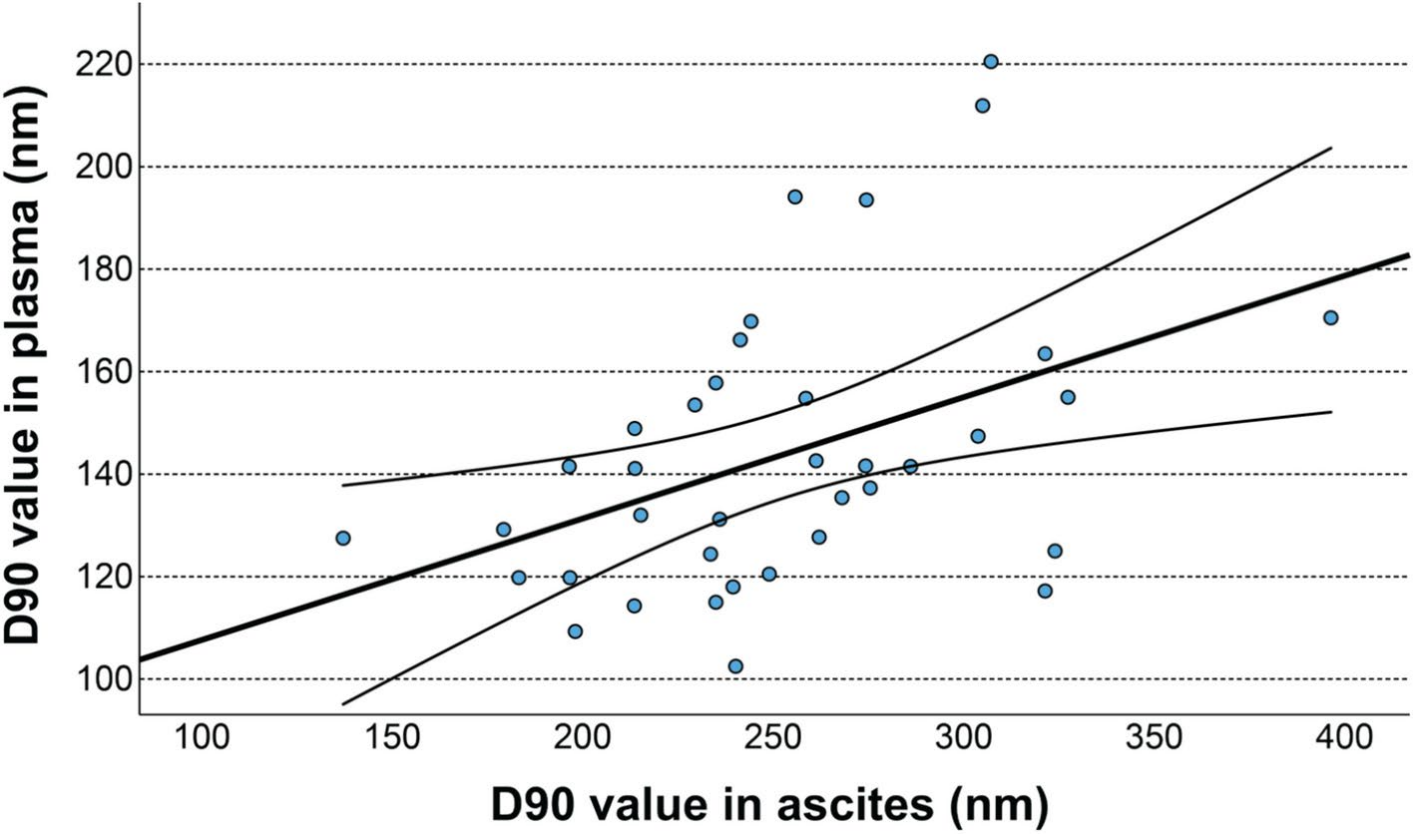
Correlation of the D90 value of the EV size distribution between ascites and plasma. The D90 percentile value represents the size below which 90% of EVs are found. The upper and lower lines on either side of the linear regression line represent the 95% confidence interval (CI).

The mean diameter of EVs was 166 nm (148.1–184.65 nm) in ascites and 83.6 nm (76.9–91.5 nm) in plasma, the modal size was 101.6 nm (93.2–127.95 nm) in ascites and 61.7 nm (59.15–66.3 nm) in plasma, D10 value was 95.9 nm (84.6–103.9 nm) in ascites and 54.9 nm (51.35–58.65 nm) in plasma, D50 value was 150 nm (128.45–166.4 nm) in ascites and 69 nm (65.3–80 nm) in plasma, D90 value was 243.8 nm (214.15–280.4 nm) in ascites and 141.1 nm (122.45–156.4 nm) in plasma. The proportion of medium/large sized (> 200 nm) EVs was 23.3% (15.55–34.7%) in ascites and 2% (1.25–3.85%) in plasma. Significant correlation of the mean EV diameter (r = 0.398, p = 0.015), D90 value (r = 0.433, p = 0.007; Fig. 1) and proportion of medium/large sized (> 200 nm) EVs (r = 0.578, p < 0.001) between ascites and plasma samples were found.

### EV characteristics in relation with clinical characteristics, treatment outcome and prognosis of patients with advanced HGSC

We investigated the relationship between EV characteristics in paired pretreatment plasma and ascites samples and various treatment outcomes, including progression-free survival (PFS) and overall survival (OS). First, we compared the EV concentration and size distribution between 22 patients with primary inoperable disease and 15 patients who underwent primary debulking surgery (PDS). No significant difference in EV concentration or size distribution was observed between these groups. Importantly, there was also no significant difference in PFS (*p* = 0.142) or OS (*p* = 0.501) between patients with PDS and patients with primary inoperable disease.

**Fig. 2:**
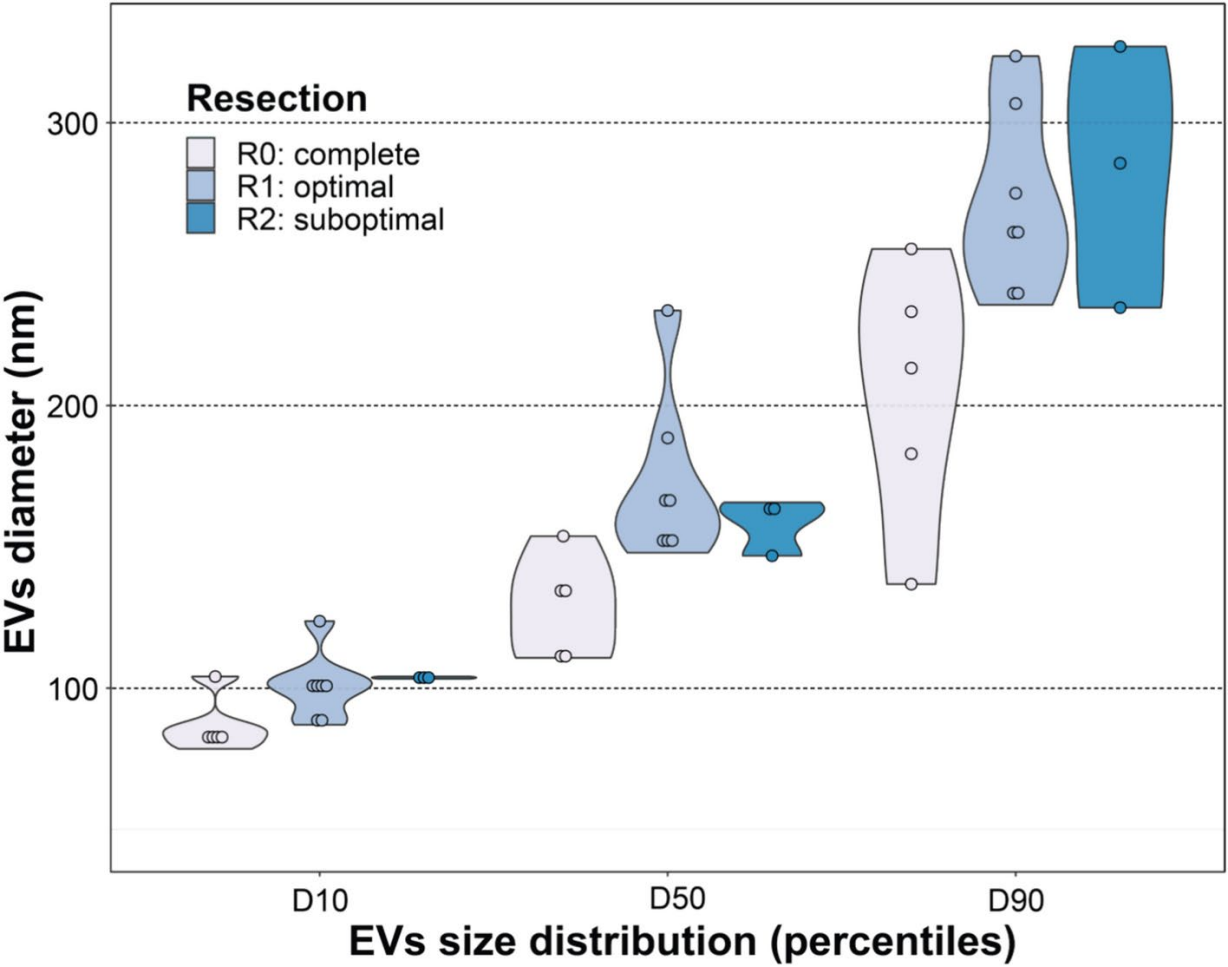
Percentile values of EVs in ascites according to resection success. D10, D50 and D90 are percentile values that represent the size below which 10%, 50% and 90% of the EV population is found.

In the 15 patients who underwent primary cytoreductive surgery, a positive correlation between resection success (residual disease R0, R1 or R2) and EV size distribution was found. We observed significant correlation of the mean EV diameter (r = 0.602, *p* = 0.018), D10 (r = 0.624, *p* = 0.013), D50 (r = 0.548, *p* = 0.035), D90 value (r = 0.664, *p* = 0.007) and proportion of medium/large-sized EVs (> 200 nm) (r = 0.517, *p* = 0.048) in ascites, but not in the plasma, with residual disease after primary cytoreductive surgery (Fig. 2).

There was no significant correlation between the total EV concentration in ascites or plasma and resection success.

Among the 22 patients with primary inoperable disease, 16 (73%) were eligible for IDS. For these 16 patients, we observed a significant negative correlation between the CRS and the D10 value of EVs in plasma (r = −0.574, *p* = 0.020). A smaller D10 value of EVs in plasma was associated with a better chemotherapy response (higher CRS).

At the conclusion of our study, only four out of the total 37 patients (11%) had no detectable disease progression, and eight patients (22%) were alive. The median follow-up time for survivors was 66 months (ranging 35–72 months). In our study, EV characteristics were not significantly correlated with PFS or OS.

To compare the predictive potential of the EV size distribution with that of the established KELIM score, the correlation of the KELIM score with resection success at PDS and with the CRS at IDS Spearman’s Rho correlation. The KELIM score was not significantly correlated with the cytoreductive outcome (r = −0.273, *p* = 0.325) or CRS (r = 0.142, *p* = 0.600).

The potential influence of patient age and body mass index (BMI) on EV size distribution was analysed. No significant correlation between age and EV size distribution in ascites or plasma was found. Additionally, no correlation between patient BMI and EV size distribution was found. Interestingly, there was a significant correlation between BMI and EV concentration in plasma (*p* = 0.002).

### EV size distribution in pretreatment ascites and plasma samples as potential predictive biomarkers for response to primary treatment in patients with advanced HGSC

We performed receiver operating characteristic (ROC) curve analysis and determined cut-off values for EV characteristics in pretreatment ascites samples to predict suboptimal (R2) resection. At the cut-off value of 103.3 nm for D10 in ascites, the sensitivity was 100%, and the specificity was 83% (AUC = 0.847, 95% CI = 0.648–1.000). At the cut-off value of 158.8 nm for D50 in ascites, the sensitivity was 67%, the specificity was 67% (AUC = 0.556, 95% CI = 0.252–0.859), and for D90, at the cut-off value of 280.4 nm, the sensitivity for the prediction of suboptimal (R2) resection was 67%, and the specificity was 83% (AUC = 0.722, 95% CI = 0.366–1.000). The results are presented in Table 2.

**Table 2:** Cut-off values, corresponding specificities, sensitivities and AUCs for identifying EV characteristics in ascites to predict suboptimal (R2) resection.

EV characteristic in ascites	Cut-off value	Sensitivity	Specificity	AUC	95% CI
D10 (nm)	103.3	100%	83%	0.847	0.648–1.000
D50 (nm)	158.8	67%	67%	0.556	0.252–0.859
D90 (nm)	280.4	67%	83%	0.722	0.366–1.000

D10, D50, and D90 are percentile values that represent the size below which 10%, 50%, and 90% of the EV population is found. Optimal cut-off values for specific EV characteristics were identified using the Youden index in ROC curves.

*EV* extracellular vesicle, *AUC* area under the curve, *CI* confidence interval.

In plasma, at the cut-off value of 49.7 nm for the D10 value, the sensitivity and specificity for predicting the complete or near-complete chemotherapy response score (CRS 3) was 100% (AUC = 1.000).

## Discussion

To improve the survival of patients with advanced HGSC, prognostic biomarkers that could help inform treatment decisions and enable personalised approach are needed^1^. The most widely used biomarker for the diagnosis of ovarian cancer OC, monitoring response to treatment and detecting disease recurrence is CA-125; however it is not always elevated in early stages and can also be elevated in benign ovarian pathology and other cancers^39^. There are no clinical biomarkers for the prediction of tumour resectability, and patients are exposed to the risks of diagnostic laparoscopy for assessing the chance for optimal cytoreduction^5^. Prognostic biomarkers for tumour resectability in advanced HGSC that could help identify patients unsuitable for primary cytoreductive surgery would enable earlier start of NACT and could improve treatment outcome. However, some patients with advanced HGSC have platinum refractory disease and do not respond to NACT, whereas in some cases, NACT might induce platinum resistance^40–43^. Therefore, prognostic biomarkers for chemotherapy response would help stratify patients for tailored therapy and improve personalised approach.

EVs that contain proteins and nucleic acids representative of the secreting cell and are accessible for minimally invasive liquid biopsy are a promising source of biomarkers for diagnostic purposes as well as for predicting response to treatment and early detection of relapse^44^. In ovarian cancer, EVs can be isolated from blood and/or ascites and have shown potential as diagnostic biomarkers^15–17^, but their potential to serve as prognostic and predictive biomarkers is still largely unexplored^45^.

In our study, pretreatment plasma and ascites samples from 37 patients with advanced HGSC were analysed. All plasma samples were taken before primary surgery and ascites samples were taken during primary cytoreductive or diagnostic operation. Nanoparticle tracking analysis (NTA) was used to determine the concentration and size distribution of EVs in paired pretreatment ascites and plasma samples. Cells release heterogeneous populations of EVs that range in size from 30 nm to 1000^46^. We use the term “small EVs” for EVs with diameter smaller than 200 nm and the term “medium/large-sized EVs” for EVs with diameter > 200 nm, as recommended by the International Society for Extracellular Vesicles^47^. We found a significant correlation between the mean EV diameter, D90 value and proportion of medium/large sized (> 200 nm) EVs in ascites and those in plasma. On the other hand, no correlation between the total EV concentration in ascites and that in plasma was found. This result is in agreement with our previous study, where the total EV concentration in plasma and ascites was determined by fluorescence-triggered flow cytometry using calcein labelling^27^.

Next, we observed the EV concentration and size distribution in ascites and plasma in relation with treatment outcome, progression-free survival (PFS) and overall survival (OS) of patients with advanced HGSC. When patients with primary inoperable disease were compared with patients who had primary cytoreductive surgery regarding EVs characteristics, no significant difference between the two groups was observed. Importantly, in our study group, there was also no significant difference in PFS or OS between patients who underwent PDS and patients with primary inoperable disease. This result is in agreement with studies that have shown that selecting patients for NACT followed by IDS based on laparoscopic evaluation of resectability prolonged PFS and did not worsen OS compared with patients who were not completely debulked with PDS (R2 resection)^48,49^. Complete resection of all macroscopic disease (R0) remains the most important prognostic factor, whether performed at PDS or after NACT^48^.

Notably, when the correlation of EV characteristics with resection success at PDS was analysed,a significant correlation of the mean EV diameter in ascites with residual disease after primary cytoreductive surgery was observed. We therefore took a closer look at the size distribution of EVs represented by percentile values and found a significant correlation between D10, D50 and D90 values, proportion of medium/large sized (> 200 nm)EVs in ascites and residual disease after primary cytoreductive surgery. Our results indicate that larger EVs in ascites correlate with worse resection success after primary cytoreductive surgery, which is one of the most important prognostic factors for the survival of patients with advanced HGSC, even in post-PARP inhibitor era^7,50^. On the other hand, no correlation of the EV concentration in plasma or ascites with resection success was observed, which is also in agreement with the results of our previously published study^27^.

Next, we observed a significant correlation between the D10 value of EVs in pretreatment plasma samples and the CRS. A smaller D10 value, which represents a size below which 10% of the EV population falls, is correlated with a better CRS. CRS is a pathological score assessed after IDS in patients treated with NACT. Patients with CRS 3 (complete or near complete tumour response) have significantly longer PFS^51^. Interestingly, there was no correlation of D10 value between ascites and plasma, which indicates that ascites is not the only source of the smallest EV population in plasma. Indeed, studies have shown that the origin, biogenesis and function of EVs in body fluids are diverse. In plasma, EVs originate from different cells and tissues. Most are derived from platelets, while an important share originates from immune and endothelial cells^52^. The potential of the size distribution of EVs to predict resistance to platinum-based chemotherapy has already been reported in our previous study on ovarian cancer cell lines, where chemoresistant cellline released EVs with larger mean, mode, D10, D50, and D90 parameters and a greater proportion of medium/large sized (> 200 nm) EVs than did the two chemosensitive cell lines^24^. Taken together, our present study and our previous studies indicate that the EV size distribution, but not the total EV concentration, is relevant for the prediction of the response to chemotherapy^24,27^.

To assess the predictive biomarker potential of the EV size distribution in ascites fluid and plasma, ROC curve analysis was performed, and cut-off values were determined. We found excellent performance of the D10 value in ascites for the prediction of suboptimal (R2) resection at PDS and outstanding performance of the D10 value in plasma for the prediction of complete or near-complete CRS (CRS 3) at IDS. The D10 value in pretreatment ascites samples might help identify patients unsuitable for primary cytoreductive surgery without exposing them to the risks of diagnostic laparoscopy, which could enable earlier start of NACT and improve treatment outcome. On the other hand, the D10 value in pretreatment plasma samples might help predict the chemotherapy response after NACT and could help stratify patients for tailored therapy and improve personalised approach.

Interestingly, in our study population, the findings indicate that the KELIM score does not demonstrate potential for predicting response to primary treatment. Specifically, no correlation between the KELIM score and either the cytoreductive outcome or CRS was observed. While many studies support its utility as a predictive tool for chemosensitivity and treatment outcome^53,54^, others suggest limitation in its predictive capacity^55^. In any case, calculating the KELIM score requires at least three CA 125 measurements within the first 100 days of chemotherapy, whereas the EV size distribution in pretreatment samples could support baseline patient stratification for personalised therapy.

Few similar studies can be found in other cancers^23,56–58^. Badovinac et al. studied plasma EV characteristics measured by NTA in patients with pancreatic ductal carcinoma (PDAC) who underwent surgery with curative intent and reported significantly higher preoperative plasma EV concentrations and smaller mean EV diameter in patients who underwent surgical resection than in patients with unresectable disease. Additionally, patients with R0 resection had higher preoperative plasma EV concentration than patients with R1/R2 resection. Furthermore, higher plasma EV concentration and smaller mean EV diameter were correlated with longer OS^23,58^.

In our study, ascites and the plasma EV concentration did not correlate with treatment outcome or survival. However, the predictive potential of the EV size distribution in pretreatment ascites and plasma samples from patients with advanced HGSC was observed. EV subpopulation heterogeneity can be categorized into (1) physical parameters, such as size, density, viscoelasticity and (2) molecular composition^59^. Given the limited sample volume, particularly for plasma, we decided to focus on adequately characterizing one aspect of heterogeneity—size. Size is one of the major determinants of EV composition. EVs of varying size from a single source can exhibit distinct biological functions. Indeed, certain functional properties of EVs, such as cell uptake, are known to differ based on their size^59,60^.

The discrepancy in relative stoichiometry of EVs is significantly amplified by size: a 150 nm EV possesses 25 times more surface area and 125 times more volume than 30 nm EV. This size difference enables the larger vesicle to potentially traffic thousands more proteins and other cargo. Zhai et al. performed a study on EVs from cancer cell lines and plasma samples of patients with liver, lung, breast and prostate cancer. They analysed specific EV protein expression levels and demonstrated that by grouping EVs into three subpopulations according to their size, the overall accuracy of discriminating between cancer types can be improved^20^. Guan et al. performed a size-dependent subproteome analysis of urinary exosomes and reported that each fraction contained unique proteins involved in different molecular and cellular processes^21^. In a study by Zheng et al., proteomic analysis of urinary exosomes divided into subpopulations of large, medium and small exosomes was performed. They reported significantly different protein content between these subpopulations, with distinct biological functions^22^.

An important finding of our study is that an increase in proportion of larger EVs in the body fluids of patients with HGSC is linked to a poorer prognosis. In aggressive cancers larger EVs are often enriched in oncogenic proteins, transporters, DNA, or RNA that promote tumour growth, metastasis, angiogenesis and immune evasion^61,62^. Larger EVs often originate from direct budding of the plasma membrane which is typically triggered by cellular stress, inflammation, hypoxia which are all hallmarks of severe disease state. Larger EVs with pro-coagulant cargo that can exacerbate processes like thrombosis, which is associated with poor outcome in cancer^63,64^.

Our current study offers a basis for further proteomic and genomic analyses of EV subpopulations. In the future, molecular profiling of size-dependent EV subpopulations in ascites and plasma from advanced HGSC patients could improve our understanding of their biological functions and biomarker potential.

The main limitation of our study is the small sample size, which reduces the statistical power and the generalisability of the findings to larger population. This limitation suggest that the findings should be interpreted with caution, and further research with larger sample size is necessary to confirm our results. Due to the small sample size, multivariate analyses including comorbidities or concurrent medications were not possible. We did, however, assess the correlation of age and BMI with EV size distribution. No significant correlation between age or BMI and EV size distribution in ascites or plasma was found. The relatively small sample size in our study is primarily due to the strict inclusion criteria for histologically confirmed HGSC, the single-centre design and rigorous standards for sample preparation and quality. HRD as a predictive biomarker for response to PARP inhibitors, was officially incorporated into the ESMO guidelines after the conclusion of our study. Consequently, not all patients who could benefit from PARP inhibitors were identified, likely impacting final treatment outcome, particularly the duration PFS. Although PARP inhibitors cannot influence the outcome of primary treatment, since they are indicated for the maintenance treatment. Additionally, our study was conducted on pretreatment samples only. The inclusion of samples during and after treatment could provide valuable information on how EV characteristics change over time in response to treatment. The mechanisms by which chemotherapy affects the composition and physical size of EVs are far from understood, but studies have shown that, in response to chemotherapy, the cargo within EVs is sorted differently and that specific cargos might be used as biomarkers to monitor the response to chemotherapy^52,65,66^. Further studies in vitro and in clinical settings are needed to understand the relationships among EV size, cargo and response to chemotherapy.

An advantage of our study is that the population while relatively small, was homogeneous in terms of disease stage and histological type. Conducting a single-center study allowed for consistency in treatment protocols and sample handling. Additionally, the statistically significant correlation between the EV size distribution and primary treatment outcome despite the low statistical power implies a very strong relationship between the observed parameters.

## Conclusions

The results of our study show for the first time that the EV size distribution in pretreatment ascites and plasma samples from patients with advanced HGSC is significantly correlated with the response to primary treatment. The mean diameter, D10, D50 and D90 values of EVs in ascites fluid were significantly correlated with resection success after primary cytoreductive surgery. Larger EVs in ascites are correlated with worse resection success. Additionally, a smaller D10 value of EVs in plasma was correlated with a better chemotherapy response at the time of IDS. ROC curve analysis revealed excellent performance of the D10 value in ascites for the prediction of suboptimal (R2) resection at PDS and outstanding performance of the D10 value in plasma for the prediction of complete or near-complete CRS (CRS 3) at IDS.

We also found a significant correlation of the mean diameter, D90 value and proportion of medium/large EVs between the ascites and plasma samples. No correlation of EV concentration or D10 percentile value between ascites and plasma was found, which indicates that the smallest EVs in plasma originate from different sources.

Taken together, our results show that the size distribution of EVs in ascites and plasma has the potential to serve as a predictive biomarker in advanced HGSC and could help stratify patients for tailored therapy and improve personalised approach in the future. Therefore, further studies are required to validate our findings.

## Data Availability

We have submitted all relevant data of our experiments to the EV-TRACK knowledgebase (EV-TRACK ID: EV240145). Should any raw data files be needed in another format they are available from the corresponding author upon reasonable request.
